# Gilteritinib versus chemotherapy in Japanese patients with *FLT3*-mutated relapsed/refractory acute myeloid leukemia

**DOI:** 10.1007/s10147-021-02006-7

**Published:** 2021-08-07

**Authors:** Naoko Hosono, Hisayuki Yokoyama, Nobuyuki Aotsuka, Kiyoshi Ando, Hiroatsu Iida, Takayuki Ishikawa, Kensuke Usuki, Masahiro Onozawa, Masahiro Kizaki, Kohmei Kubo, Junya Kuroda, Yukio Kobayashi, Takayuki Shimizu, Shigeru Chiba, Miho Nara, Tomoko Hata, Michihiro Hidaka, Shin-Ichiro Fujiwara, Yoshinobu Maeda, Yasuyoshi Morita, Mikiko Kusano, Qiaoyang Lu, Shuichi Miyawaki, Erhan Berrak, Nahla Hasabou, Tomoki Naoe

**Affiliations:** 1 Department of Hematology and Oncology, Faculty of Medical Sciences, University of Fukui, Fukui, 910-1193 Japan; 2 Sendai Medical Center, Sendai, Japan; 3 Japanese Red Cross Narita Hospital, Narita, Japan; 4 Tokai University School of Medicine, Isehara, Japan; 5 NHO Nagoya Medical Center, Nagoya, Japan; 6 Kobe City Medical Center General Hospital, Hyogo, Japan; 7 NTT Medical Center Tokyo, Tokyo, Japan; 8 Hokkaido University, Sapporo, Japan; 9 Saitama Medical Center, Saitama Medical University, Kawagoe, Japan; 10 Aomori Prefectural Central Hospital, Aomori, Japan; 11 Kyoto Prefectural University of Medicine, Kyoto, Japan; 12 International University of Health and Welfare (IUHW), Mita Hospital, Tokyo, Japan; 13 Keio University School of Medicine, Tokyo, Japan; 14 University of Tsukuba, Tsukuba, Japan; 15 Akita University, Akita, Japan; 16 Nagasaki University, Nagasaki, Japan; 17 Kumamoto Medical Center, Kumamoto, Japan; 18 Jichi Medical University, Shimotsuke, Japan; 19 Okayama University Hospital, Okayama, Japan; 20 Kindai University, Osaka, Japan; 21 Astellas Pharma, Inc., Tokyo, Japan; 22 Astellas Pharma US, Inc., Northbrook, IL USA; 23 Tokyo Metropolitan Ohtsuka Hospital, Tokyo, Japan; 24 Present Address: Tohoku University, Sendai, Japan

**Keywords:** Acute myeloid leukemia, FLT3 inhibitor, FLT3 mutations

## Abstract

**Background:**

Until recently, no effective targeted therapies for *FLT3*-mutated (*FLT3*^mut+)^ relapsed/refractory (R/R) acute myeloid leukemia (AML) were available in Japan. The FLT3 inhibitor, gilteritinib, was approved in Japan for patients with *FLT3*^mut+^ R/R AML based on the phase 3 ADMIRAL trial, which demonstrated the superiority of gilteritinib over salvage chemotherapy (SC) with respect to overall survival (OS; median OS, 9.3 vs 5.6 months, respectively; hazard ratio, 0.64 [95% confidence interval 0.49, 0.83]; *P* < 0.001).

**Methods:**

We evaluated the Japanese subgroup (*n* = 48) of the ADMIRAL trial, which included 33 patients randomized to 120-mg/day gilteritinib and 15 randomized to SC.

**Results:**

Median OS was 14.3 months in the gilteritinib arm and 9.6 months in the SC arm. The complete remission/complete remission with partial hematologic recovery rate was higher in the gilteritinib arm (48.5%) than in the SC arm (13.3%). After adjustment for drug exposure, fewer adverse events (AEs) occurred in the gilteritinib arm than in the SC arm. Common grade ≥ 3 AEs related to gilteritinib were febrile neutropenia (36%), decreased platelet count (27%), and anemia (24%).

**Conclusion:**

Findings in Japanese patients are consistent with those of the overall ADMIRAL study population.

## Introduction

Acute myeloid leukemia (AML) is the most common leukemia among Japanese adults, and accounts for approximately 70% of all myeloid leukemias [1, 2]. Patients with relapsed AML have a poor prognosis, with a 5-year survival rate of 10–11% [3, 4]. Activating mutations in *fms-like tyrosine kinase 3* (*FLT3*) occur in approximately 30% of patients with AML [5, 6], primarily as internal tandem duplication (*FLT3*-ITD) mutations in the juxtamembrane region (~ 20–25%) and missense point mutations in the tyrosine kinase domain (*FLT3*-TKD; ~ 5–7%) [5, 7–11]. In AML, *FLT3*-ITD mutations confer a strong negative impact on survival[12–14]; *FLT3*-TKD point mutations have been implicated in resistance to FLT3 inhibitors [15, 16].

Several FLT3 tyrosine kinase inhibitors (TKIs) are under development or have been approved for the treatment of *FLT3*-mutated (*FLT3*^mut+^) AML. First-generation multi-targeted FLT3 TKIs (sunitinib, sorafenib, midostaurin, lestaurtinib, ponatinib) [17] lacked specificity for *FLT3*-ITD, resulting in short-lived antileukemic activity, especially as single agents [18–21]. Second-generation FLT3 TKIs (gilteritinib and quizartinib) boasting higher potency and greater specificity for FLT3 were subsequently developed [17]. While quizartinib demonstrated single-agent activity in relapsed/refractory (R/R) *FLT3-*ITD–positive (*FLT3*-ITD +) AML patients [22], treatment-emergent *FLT3*-TKD mutations leading to drug resistance [15] and myelosuppression due to quizartinib’s effects on c-kit [23] are of concern.

Gilteritinib is a highly selective, oral FLT3 inhibitor with activity against both *FLT3*-ITD and -TKD mutations with only weak activity against c-Kit [24, 25]. The phase 1/2 CHRYSALIS study demonstrated that single-agent gilteritinib had a favorable safety profile and, at ≥ 80-mg/day doses, induced ≥ 90% inhibition of FLT3 autophosphorylation with a composite complete remission (CRc) rate of 41% in patients with R/R *FLT3*^mut+^ AML [26]. A starting dose of 120 mg/day was recommended for further study [26].

Gilteritinib was the first FLT3 TKI approved by the Ministry of Health Labor and Wealth (MHLW) in Japan for patients with R/R *FLT3*^mut+^ AML [27]. Approval was based on interim results from the phase 3 ADMIRAL study (NCT02421939) of gilteritinib versus salvage chemotherapy (SC) in patients with R/R *FLT3*^mut+^ AML, where 40 of 142 (28%) patients in the gilteritinib arm had achieved complete remission (CR) or CR with partial hematologic recovery (CRh) [27]. Final results from ADMIRAL demonstrated significantly longer overall survival (OS) with gilteritinib than with SC (9.3 vs 5.6 months, respectively; hazard ratio [HR] for death, 0.64; 95% confidence interval [CI] 0.49, 0.83; *P* < 0.001) [28].

As treatment efficacy and safety may vary by ethnicity, evaluation of gilteritinib in Japanese patients with R/R *FLT3*^mut+^ AML is warranted. The safety and antitumor effects of gilteritinib were reported in a phase 1 study of 24 Japanese patients with R/R AML; however, only 3 *FLT3*^mut+^ Japanese patients had received ≥ 80-mg gilteritinib [29]. The ADMIRAL trial included 54 R/R *FLT3*^mut+^ AML patients from Asian countries, with most (89%; 48 of 54) being Japanese. A subgroup analysis was conducted to confirm the clinical risk–benefit profile of gilteritinib in Japanese patients enrolled in the ADMIRAL trial.

## Materials and methods

### Trial design and oversight

This randomized phase 3, open-label study (NCT02421939; ADMIRAL) was conducted at 107 centers in 14 countries [28]. Japanese patients were enrolled at 20 sites across Japan. The trial was reviewed and approved by site-specific institutional review boards or ethics committees and conducted according to the principles of the Declaration of Helsinki. All patients provided written informed consent at enrollment. All authors had access to study data and have ascertained its accuracy and adherence to the study protocol.

### Patients

Complete details of the ADMIRAL population are provided in the primary publication [28]. Adults with AML were eligible if they were refractory to one or two cycles of conventional anthracycline-containing induction therapy or were experiencing hematologic relapse after first CR. Patients ineligible for anthracycline-containing induction regimens could participate if they had completed ≥ 1 cycle of alternative standard therapy judged as the optimum choice to induce remission. At enrollment, patients’ marrow and blood were screened for *FLT3* mutations by a central laboratory. Enrollment based on local *FLT3* mutation testing was permitted in patients with rapidly proliferative disease. Patients were required to have either *FLT3*-ITD or *FLT3*-TKD D835 or I836 mutations (Invivoscribe; San Diego, CA) using a polymerase chain reaction-based assay modeled on published methods (LeukoStrat^®^ CDx) [30].

### Randomization and treatments

A brief description of the ADMIRAL study design, randomization, and treatment are described (Supplement, Figure S1), with further details in the primary publication [28]. Briefly, enrolled patients were randomized 2:1 to receive 28-day cycles of once-daily gilteritinib (120 mg) or preselected high- or low-intensity SC. High- or low-intensity SC regimens were selected by study investigators before randomization from the following options: high-intensity SC: mitoxantrone, etoposide, and cytarabine (MEC)[31], or fludarabine, cytarabine, granulocyte-colony stimulating factor, and idarubicin (FLAG-IDA)[32]; or low-intensity SC: low-dose cytarabine (LoDAC) or azacitidine (AZA). High-intensity chemotherapy was administered for one to two cycles; gilteritinib or low-intensity SC were administered until documented lack of clinical benefit, intolerance, or other protocol-defined discontinuation criterion [28].

### Endpoints and assessments

The co-primary endpoints were OS and the rates of CR/CRh, where CRh was defined as CR with a platelet count of > 0.5 Gi/L and an absolute neutrophil count of > 0.5 Gi/L. Key secondary endpoints were event-free survival (EFS) and CR rate, where EFS was defined as the time from the date of randomization until the date of documented relapse (excludes relapse after partial remission), treatment failure, or death. The CR/CRh rate was evaluated in the first interim analysis in the gilteritinib arm only and was also summarized in the final analysis for both treatment arms. Overall survival, EFS, CR rate, and other response outcomes were evaluated in the final analysis. Treatment response was assessed using the modified International Working Group criteria [33]; best response was captured at any postbaseline visit. Treatment-emergent adverse events (TEAEs) were graded according to the Common Terminology Criteria for Adverse Events (v4.03). The exposure-adjusted incidence of TEAEs per patient-year (PY) was calculated by dividing exposure duration in years by the number of patients in each treatment arm. Postbaseline (i.e., 29 days after first dose until the last dose) transfusion status was evaluated in patients on gilteritinib therapy for ≥ 84 days; transfusion independence was achieved if no red blood cell/platelet transfusions were administered for 56 consecutive days during the postbaseline period. The incidence and duration of hospitalization were also evaluated.

### Statistical analysis

Complete details on statistical analyses in the ADMIRAL trial have been previously published [28]. The CR/CRh rate in the gilteritinib arm was assessed at the first planned interim analysis when approximately 141 patients in the intention-to-treat (ITT) population had reached 112 days (four treatment cycles) after randomization or the first dose; interim evaluation of CR/CRh rate had no impact on trial conduct [28].

The Japanese subgroup was analyzed similarly to the overall ITT population. The Kaplan–Meier method combined with the Greenwood formula was used to determine OS and EFS and corresponding 95% CIs. As the trial was designed to test hypotheses in the overall ITT population, it was not powered to detect cross-arm differences in this subgroup analysis.

## Results

### Patient disposition

**Fig. 1:**
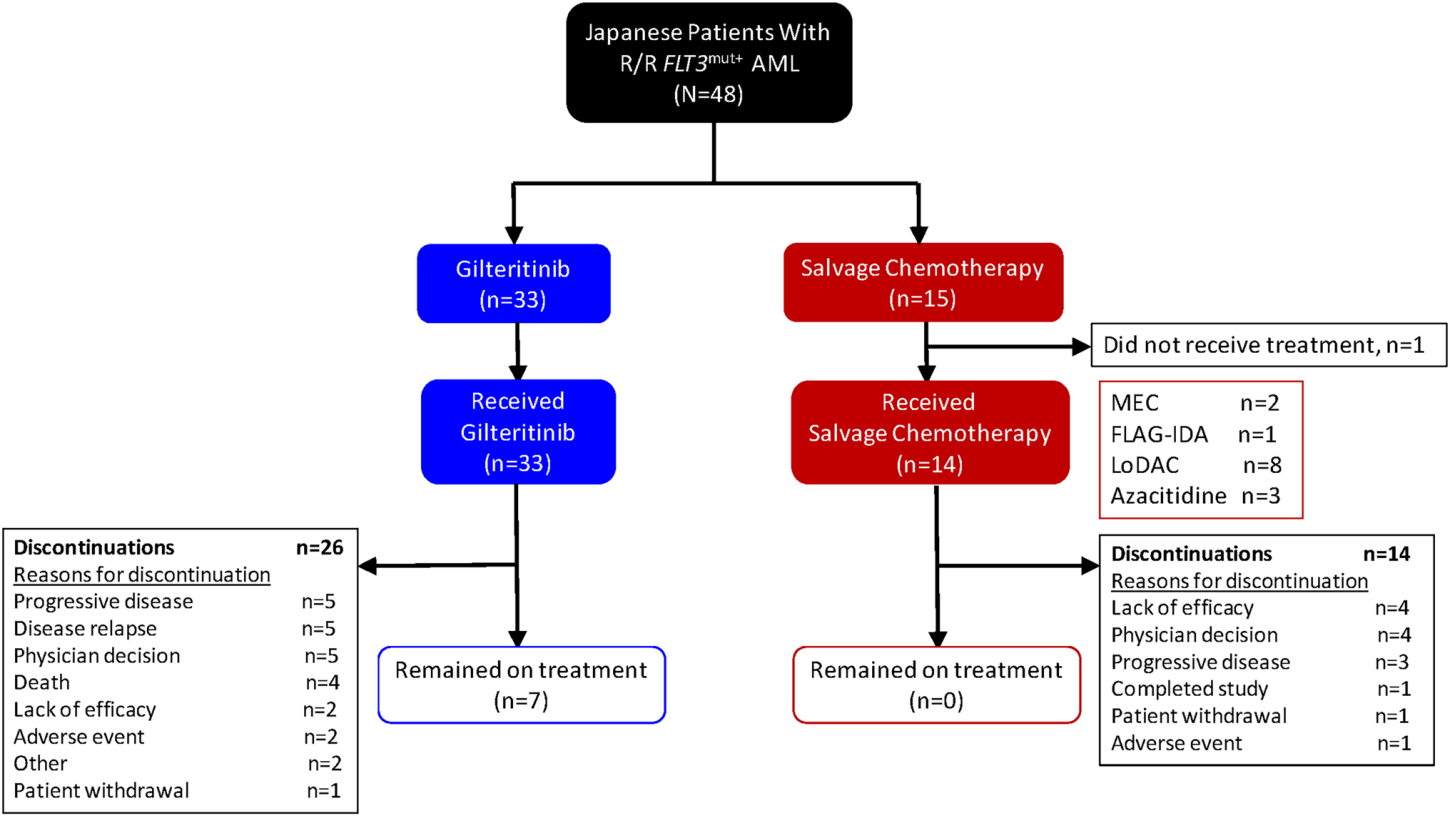
Patient disposition: Japanese ITT population. *AML* acute myeloid leukemia, *FLAG-IDA* fludarabine, cytarabine, and idarubicin plus granulocyte colony-stimulating factor, *ITT* intention-to-treat, *LoDAC* low-dose cytarabine, *MEC* mitoxantrone, etoposide, and cytarabine, *mut + * mutated, *R/R* relapsed or refractory

Of the 625 patients screened from October 2015 to February 2018, 371 (ITT population) were randomized to gilteritinib (*n* = 247) or SC (*n* = 124), which included 48 Japanese patients (gilteritinib, *n* = 33; SC, *n* = 15) (Fig. 1). At the time of primary analysis (September 17, 2018), 24 of 48 (50%) Japanese patients remained alive (*n* = 15/33, gilteritinib; *n* = 9/15, SC) and 7 remained on gilteritinib therapy. In the Japanese subgroup, 79% (*n* = 11/14) of patients in the SC arm received low-intensity chemotherapy compared with 38% (*n* = 41/109) of SC-treated patients in the overall safety analysis set (SAF) population.

### Demographic and baseline characteristics

As shown in Table 1, 46% (*n* = 22/48) of patients had relapsed AML (gilteritinib, 52% [*n* = 17/33]; SC, 33% [*n* = 5/15]) and 54% (*n* = 26/48) had primary refractory disease (gilteritinib, 48% [*n* = 16/33]; SC, 67% [*n* = 10/15]). The proportion of Japanese patients aged ≥ 65 years was higher in the SC arm (*n* = 13/15; 87%) than in the gilteritinib arm (*n* = 15/33; 45%). Japanese patients had lower body weight (mean ± standard deviation [SD]: 52.91 ± 11.10 kg) compared with the overall ITT population (mean ± SD: 71.82 ± 20.25 kg).

**Table 1:** Patient demographics and disposition (Japan ITT population; *N* = 48)

Characteristic	Gilteritinib (*n* = 33)	Salvage chemotherapy (*n* = 15)	Total (*N* = 48)
Median age, years (range)	60.0 (22–84)	69.0 (28–79)	67.5 (22–84)
Sex, *n* (%)			
Male	14 (42)	6 (40)	20 (42)
Female	19 (58)	9 (60)	28 (58)
Age group^a^, *n* (%)			
< 65 years	18 (55)	2 (13)	20 (42)
≥ 65 years	15 (45)	13 (87)	28 (58)
ECOG performance status, *n* (%)			
0–1	30 (91)	14 (93)	44 (92)
≥ 2	3 (9)	1 (7)	4 (8)
Central *FLT3* mutation status^a^, *n* (%)			
* FLT3*-ITD only	29 (88)	15 (100)	44 (92)
* FLT3*-TKD only	3 (9)	0	3 (6)
* FLT3*-ITD and *FLT3*-TKD	1 (3)	0	1 (2)
AML type, *n* (%)			
De novo	31 (94)	15 (100)	46 (96)
Secondary	2 (6)	0	2 (4)
Cytogenetic risk status^a^, *n* (%)			
Favorable	2 (6)	0	2 (4)
Intermediate	20 (61)	13 (87)	33 (69)
Unfavorable	2 (6)	1 (7)	3 (6)
Other/unknown	9 (27)	1 (7)	10 (21)
Response to first-line therapy, *n* (%)			
Primary refractory AML without HSCT	16 (48)	10 (67)	26 (54)
Relapse ≤ 6 months after allogeneic HSCT	2 (6)	0	2 (4)
Relapse > 6 months after allogeneic HSCT	2 (6)	0	2 (4)
Relapse ≤ 6 months after CRc and no HSCT	7 (21)	3 (20)	10 (21)
Relapse > 6 months after CRc and no HSCT	6 (18)	2 (13)	8 (17)
Prior use of a FLT3 inhibitor, *n* (%)			
Yes	0	0	0
No	33 (100)	15 (100)	48 (100)
Previous HSCT, *n* (%)			
Yes	4 (12)	0	4 (8)
No	29 (88)	15 (100)	44 (92)
Preselected chemotherapy^a^, *n* (%)			
High-intensity	16 (48)	3 (20)	19 (40)
Low-intensity	17 (52)	12 (80)	29 (60)

Percentages were rounded to the nearest whole number

*AML* acute myeloid leukemia, *CRc* complete composite remission, *ECOG* Eastern Cooperative Oncology Group, *FLT3* *fms*-like tyrosine kinase 3, *HSCT* hematopoietic stem cell transplantation, *ITD* internal tandem duplication, *TKD* tyrosine kinase domain

^a^Percentages may not add up to 100 due to rounding

### Study drug exposure and dose modifications

The median duration of exposure to gilteritinib in the Japanese subgroup was 5.1 months

(154 days) (Table 2), which was longer than that reported for the overall SAF population (4.1 months); the median duration of exposure to SC was the same in both populations (0.9 months). Overall, 33% of gilteritinib-treated patients (*n* = 11/33) in the Japanese subgroup had dose increases to 200 mg/day (Table 2), which was similar to the overall SAF population (32%). Gilteritinib dose reductions were slightly higher in the Japanese subgroup (39%; *n* = 13/33) than in the overall SAF population (31%); dose interruptions were also higher in the Japanese subgroup (67% vs 50%, respectively).

**Table 2:** Study drug exposure and dose modifications (Japan SAF; *N* = 47)

Parameter	Gilteritinib (*n* = 33)	Salvage chemotherapy (*n* = 14)			
		MEC (*n* = 2)	FLAG-IDA (*n* = 1)	Azacitidine (*n* = 3)	LoDAC (*n* = 8)
Median duration of exposure, days (range)	154 (37–595)	28 (28–28)	28 (28–28)	28 (28–111)	28 (28–28)
Duration of exposure, *n* (%)					
≥ 6 to < 28 days	0	0	0	0	0
≥ 28 to < 84 days	9 (27)	2 (100)	1 (100)	2 (67)	8 (100)
≥ 84 to < 168 days	8 (24)	0	0	1 (33)	0
≥ 168 days	16 (48)	0	0	0	0
Median number of treatment cycles (range)	6 (2–22)	1 (1–1)	1 (1–1)	1 (1–4)	1 (1–1)
Dose modifications, *n* (%)					
Increases*	11 (33)	0	0	0	0
Decreases*	13 (39)	0	0	0	0
Interruptions	22 (67)	0	0	1 (33)	0

Percentages were rounded to the nearest whole number

*FLAG-IDA* fludarabine, cytarabine, idarubicin, and granulocyte colony-stimulating factor, *LoDAC* low-dose cytarabine, *MEC* mitoxantrone, etoposide, and cytarabine, *SAF* safety analysis set

*One patient who had a dose increase from 120 to 200 mg followed by a dose decrease to 120 mg and a subsequent dose decrease from 120 to 80 mg

### Survival outcomes

**Fig. 2:**
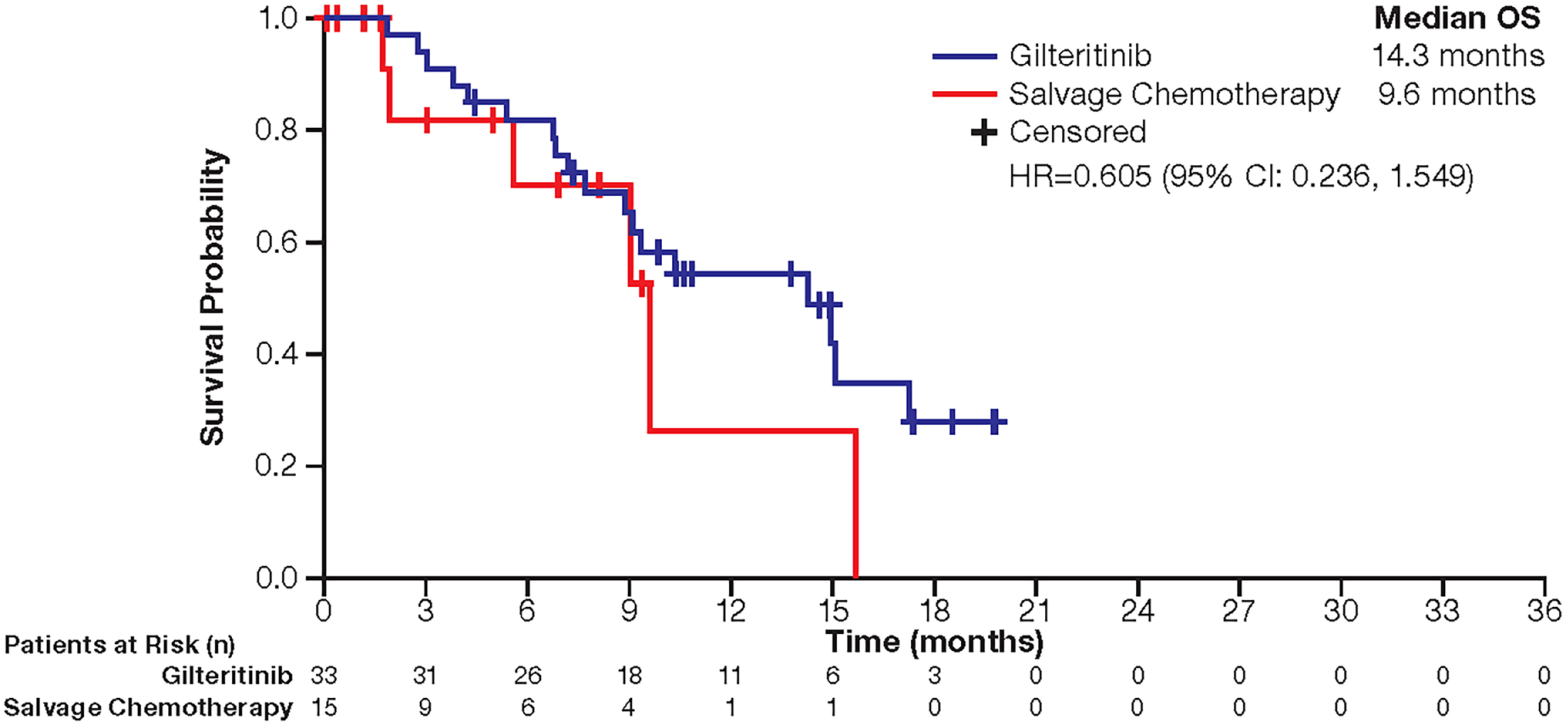
Overall survival (Japan ITT population; *N* = 48). *HR* hazard ratio, *ITT* intention-to-treat, *OS* overall survival

In the overall ITT population, median OS was 9.3 months in the gilteritinib arm and 5.6 months in the SC arm; 1-year survival rates were 37% for patients who received gilteritinib compared with 17% for patients who received SC. In the Japanese subgroup, the median OS was 14.3 months in gilteritinib-treated patients and 9.6 months in patients treated with SC (Fig. 2); 1-year survival rates were 54.3% in the gilteritinib arm versus 26.3% in the SC arm.

### Response outcomes

In the overall ITT population, the rate of CR/CRh was higher in the gilteritinib arm than in the chemotherapy arm (34.0% vs 15.3%, respectively), as was the CR rate (21.1% vs 10.5%, respectively). Similarly, CR/CRh rates in the Japanese subgroup were also higher in the gilteritinib arm (48.5%) than in the SC arm (13.3%) (Table 3). Rates of CR/CRh before HSCT in the Japanese subgroup gilteritinib and SC arms were 30.3% and 13.3%, respectively, and were comparable to the overall ITT population (gilteritinib, 26.3%; SC, 15.3%); CR rates (gilteritinib, 24.2%; SC, 6.7%) in the Japanese subgroup were also similar to the overall ITT population (gilteritinib, 21.1%; SC, 10.5%). Median durations of CR/CRh or CR in the gilteritinib arm were not reached; the median duration of CRc in the gilteritinib arm was 9.2 months. Median durations of CR/CRh, CR, and CRc were not evaluable in the SC arm. In the overall ITT population, median durations of CR, CR/CRh, and CRc in the gilteritinib arm were 14.8, 11.0, and 4.6 months, respectively; median durations of CR and CR/CRh in the SC arm were both 1.8 months; the median duration of CRc was not evaluable.

**Table 3:** Response outcomes (Japan ITT Population; *N* = 48)

Response parameter	Gilteritinib (*n* = 33)	Salvage chemotherapy (*n* = 15)
CR, *n* (%)	8 (24.2)	1 (6.7)
CRp, *n* (%)	3 (9.1)	0
CRi, *n* (%)	8 (24.2)	2 (13.3)
CRh, *n* (%)	8 (24.2)	1 (6.7)
CR/CRh, *n* (%)	16 (48.5)	2 (13.3)
CRc^a^, *n* (%)	19 (57.6)	3 (20.0)
PR, *n* (%)	5 (15.2)	1 (6.7)
NR, *n* (%)	9 (27.3)	5 (33.3)
ORR^b^, *n* (%)	24 (72.7)	4 (26.7)
NE, *n* (%)	0	6 (40.0)

*CR* complete remission, *CRc* composite complete remission, *CRh* complete remission with partial hematologic recovery, *CRi* complete remission with incomplete hematologic recovery, *CRp* complete remission with incomplete platelet recovery, *ITT* intention-to-treat, *NE* not evaluable, *NR* no response, *ORR* overall response rate, *PR* partial remission

^a^Defined as CR plus CRi plus CRp

^b^Defined as CRc plus PR

### Transplantation rates

In the overall ITT population, more patients assigned to gilteritinib (*n* = 63/247; 25.5%) than those assigned to SC (*n* = 19/124; 15.3%) underwent HSCT during the study. In the Japanese subgroup, 12 patients (36.4%) assigned to gilteritinib and 2 (13.3%) assigned to SC underwent HSCT. Nine Japanese patients who underwent HSCT after initial gilteritinib treatment resumed gilteritinib after transplantation.

### Safety and tolerability

Most patients in the Japanese SAF (*N* = 47) experienced TEAEs (Table 4; gilteritinib, 100% [*n* = 33/33]; SC, 93% [*n* = 13/14]). Drug-related TEAEs occurred in 88% of patients treated with gilteritinib and in 71% of patients treated with SC. Drug-related TEAEs leading to gilteritinib discontinuation occurred in three patients (hyperglycemia, intestinal ischemia, sepsis, back pain, depressed consciousness [*n* = 1]; cholecystitis [*n* = 1]; pneumonia [*n* = 1]). Drug-related TEAEs leading to discontinuation of SC occurred in one patient (febrile neutropenia, diabetes mellitus, and delirium). Serious drug-related TEAEs occurring in ≥ 2 gilteritinib-treated patients were febrile neutropenia (*n* = 3), increased alanine aminotransferase (ALT; *n* = 3), increased aspartate aminotransferase (AST; *n* = 3), and pneumonia (*n* = 2). Five gilteritinib-treated patients died from TEAEs: AML progression (*n* = 2); intestinal ischemia, sepsis, depressed consciousness (*n* = 1); pancreatitis (*n* = 1); and pneumonia (*n* = 1). Two deaths stemmed from drug-related TEAEs (intestinal ischemia, sepsis, depressed consciousness [*n* = 1]; pneumonia [*n* = 1]).

**Table 4:** Overview of Treatment-Emergent Adverse Events (Japan SAF; *N* = 47)

TEAEs	Gilteritinib (*n* = 33)		Salvage chemotherapy (*n* = 14)	
	All TEAEs, *n* (%)	TEAEs/PY	All TEAEs, *n* (%)	TEAEs/PY
Any TEAE	33 (100)	53.84	13 (93)	93.08
Drug-related TEAE	29 (88)	19.73	10 (71)	51.54
Serious TEAE	24 (73)	4.49	1 (7)	0.77
Drug-related serious TEAE	13 (39)	2.05	1 (7)	0.77
TEAE leading to death	5 (15)	0.38	0	0
Drug-related TEAE leading to death	2 (6)	0.22	0	0
TEAE leading to discontinuation	4 (12)	0.43	1 (7)	2.31
Drug-related TEAE leading to discontinuation	3 (9)	0.38	1 (7)	2.31
Grade ≥ 3 TEAE	32 (97)	14.05	13 (93)	45.38
Drug-related grade ≥ 3 TEAE	25 (76)	6.27	9 (64)	26.92

Percentages were rounded to the nearest whole number

*PY* patient-year, *SAF* safety analysis set, *TEAE* treatment-emergent adverse event

Gilteritinib was generally well tolerated in this Japanese subgroup. Although gilteritinib exposure was approximately five times longer than SC exposure, the exposure-adjusted incidence of TEAEs was lower with gilteritinib (53.84/PY) than with SC (93.08/PY; Table 4), including all drug-related TEAEs leading to discontinuation (gilteritinib, 0.38/PY; SC, 2.31/PY) and grade ≥ 3 drug-related TEAEs (gilteritinib, 6.27/PY; SC, 26.92/PY). In the Japanese subgroup, incidences of TEAEs (100%) and deaths (55%) in gilteritinib-treated patients were similar to those observed in the overall SAF population (100% and 69%, respectively).

The most common TEAEs in gilteritinib-treated patients were febrile neutropenia (55%), increased AST (52%), increased ALT (46%), anemia (39%), constipation (39%), increased blood creatinine phosphokinase (39%), decreased platelet count (39%), and nausea (36%) (Table 5). Most common TEAEs in patients treated with SC were anemia (57%), decreased platelet count (50%), and decreased white blood cell count (36%). Higher incidences of grade ≥ 3 febrile neutropenia (55%) and neutropenia (12%) were seen with gilteritinib versus SC (29% and 7%, respectively). Lower incidences of grade ≥ 3 anemia (33%) and thrombocytopenia (9%) were observed with gilteritinib versus SC (57% and 21%, respectively).

**Table 5:** Treatment-emergent adverse events occurring in ≥ 30% of patients in any treatment arm (Japan SAF; *N* = 47)

TEAE	Gilteritinib (*n* = 33)		Salvage chemotherapy (*n* = 14)	
	All TEAEs, *n* (%)	TEAEs/PY	All TEAEs, *n* (%)	TEAEs/PY
Febrile neutropenia	18 (55)	1.51	4 (29)	3.85
Increased aspartate aminotransferase	17 (52)	3.24	2 (14)	1.54
Increased alanine aminotransferase	15 (46)	2.38	1 (7)	0.77
Anemia	13 (39)	1.08	8 (57)	6.15
Constipation	13 (39)	0.97	3 (21)	2.31
Increased blood creatine phosphokinase	13 (39)	3.19	0	0
Nausea	12 (36)	1.03	1 (7)	0.77
Decreased platelet count	13 (39)	1.41	7 (50)	16.15
Pyrexia	11 (33)	0.92	1 (7)	0.77
Increased blood lactate dehydrogenase	10 (30)	0.54	1 (7)	0.77
Decreased white blood cell count	7 (21)	1.35	5 (36)	4.62

Percentages were rounded to the nearest whole number

*PY* patient-year, *SAF* safety analysis set, *TEAE* treatment-emergent adverse event

Most common grade ≥ 3 drug-related TEAEs observed with gilteritinib treatment in the Japanese subgroup were febrile neutropenia (36%), decreased platelet count (27%), and anemia (24%) (Table 6). When comparing the Japanese subgroup to all other ADMIRAL patients, no clinically significant TEAEs were unique to Japanese patients.

**Table 6:** Grade ≥ 3 drug-related treatment-emergent adverse events occurring in > 5% of patients in any treatment arm (SAF; *N* = 355)

Grade ≥ 3 drug-related TEAE	Japanese patients (*n* = 47)		All other patients (*n* = 308)	
	Gilteritinib (*n* = 33)	Salvage chemotherapy (*n* = 14)	Gilteritinib (*n* = 213)	Salvage chemotherapy (*n* = 95)
Febrile neutropenia	12 (36)	3 (21)	26 (12)	17 (18)
Decreased platelet count	9 (27)	4 (29)	21 (10)	10 (11)
Anemia	8 (24)	6 (43)	40 (19)	15 (16)
Decreased white blood cell count	6 (18)	4 (29)	20 (9)	10 (11)
Decreased neutrophil count	6 (18)	2 (14)	18 (9)	7 (7)
Increased alanine aminotransferase	6 (18)	0	13 (6)	2 (2)
Increased aspartate aminotransferase	6 (18)	0	14 (7)	1 (1)
Neutropenia	4 (12)	1 (7)	17 (8)	7 (7)
Increased blood creatine phosphokinase	2 (6)	0	3 (1)	0
Drug eruption	2 (6)	1 (7)	0	0
Pneumonia	2 (6)	1 (7)	5 (2)	3 (3)
Thrombocytopenia	2 (6)	2 (14)	28 (13)	9 (10)
Decreased appetite	0	2 (14)	0	0
Tumor lysis syndrome	0	2 (14)	0	0

Percentages were rounded to the nearest whole number

*SAF* safety analysis set, *TEAE* treatment-emergent adverse event

Overall, seven gilteritinib-treated patients and two SC-treated patients in the Japanese subgroup had a maximum postbaseline mean Fridericia-corrected QT interval (QTcF) increase of > 30 ms; two patients treated with gilteritinib had a maximum postbaseline mean QTcF increase of > 60 ms. One case of prolonged QTcF occurred with gilteritinib. As no maximum postbaseline QTcF increases of > 500 ms occurred, no gilteritinib dose reductions or interruptions were required. No cases of Torsade de Pointes were observed in the entire SAF population.

### Transfusion status and hospitalization in patients treated with gilteritinib

Among gilteritinib-treated Japanese patients, 22 were transfusion-dependent (TD) and 11 were transfusion-independent (TI) at baseline. Six of 22 TD patients (27%) at baseline became TI during the postbaseline period. Eight of 11 (73%) TI patients at baseline remained TI postbaseline.

Hospitalization rates during gilteritinib therapy were 94% in the Japanese subgroup and 85% in the overall ITT population; most hospitalizations (~ 96%) in both populations were outside the intensive care unit. While Japanese patients had longer hospitalization duration (median, 133 days; range 10–497 days) than the overall ITT population (median, 28 days; range 1–214 days), hospitalizations due to TEAEs were less frequent (55% vs 76%, respectively).

## Discussion

The utility of targeting *FLT3* mutations in AML has gained momentum based on findings from clinical trials [22, 25, 26, 28, 34] and the approval of FLT3-targeted agents [27, 35]. Results from the QuANTUM-R and ADMIRAL studies provide compelling evidence that targeting FLT3 improves response rate compared with SC in patients with R/R *FLT3*^mut+^ AML [22, 28]. However, it is important to note that the ADMIRAL trial enrolled patients with either *FLT3*-ITD and/or *FLT3*-TKD mutations due to gilteritinib’s activity against both mutation types, and included patients who had relapsed within or after 6 months following initial therapy [28]. The QuANTUM-R trial only enrolled patients with *FLT3*-ITD mutations who had relapsed within 6 months of initial therapy [22]. While findings from single-arm studies of gilteritinib and quizartinib in Japanese R/R AML patients have been reported [29, 36], this is the first comparative evaluation of gilteritinib versus conventional SC in a Japanese R/R AML cohort.

In the overall study population, patients randomized to gilteritinib had significantly longer OS and numerically higher rates of CR/CRh compared with patients randomized to SC [28]. The Japanese subgroup exhibited a similar trend, with numerically longer OS and improved CR/CRh rates compared with SC (49% vs 13%, respectively). In contrast to the overall study population (40%), most Japanese patients (60%) had been preselected for low-intensity chemotherapy. Overall, 79% of Japanese patients in the SC arm received low-intensity chemotherapy and none of these patients achieved CR/CRh. Findings related to safety and tolerability in the Japanese subgroup were generally consistent with those of the overall study population [28].

Exposure to gilteritinib was longer in Japanese patients (median, 5.1 months) than in the overall study population (median, 4.1 months) and a greater proportion of Japanese patients required dose reductions (39%) or interruptions (67%) compared with the overall study population (31% and 50%, respectively). The median duration of hospitalization was also longer in the Japanese subgroup (133 days) compared with the overall study population (28 days), which may partially stem from the fact that extended hospitalization does not necessarily increase medical expenses for Japanese patients eligible for the high-cost medical expense benefit issued by the Japan MHLW. Notably, oral gilteritinib therapy allows for outpatient treatment, which could potentially lower treatment costs and improve quality of life.

A phase 2 study of quizartinib in Japanese patients with R/R *FLT3*-ITD + AML reported a similar rate of CRc (54%) to that observed with gilteritinib (58%) in the Japanese subgroup from the ADMIRAL trial [36]. However, CRi was the most frequent response (48%) with quizartinib in the Japanese phase 2 study, with no patients achieving CR [36]. In contrast, 24% of Japanese patients achieved CR with gilteritinib in the ADMIRAL trial. A higher incidence of QT prolongation (35%) was observed with quizartinib [36] compared with gilteritinib (< 10%), whereas liver enzyme (AST/ALT) elevations occurred more frequently with gilteritinib (46–52%) than with quizartinib (< 10–11%). Although Japanese patients treated with gilteritinib in the ADMIRAL trial had a higher incidence of grade ≥ 3 febrile neutropenia (55%) than those who received quizartinib in the Japanese phase 2 study (43%), rates of grade ≥ 3 anemia (33% vs 27%) and thrombocytopenia (9% vs 11%) were similar [36].

As is the nature of subgroup analyses, this analysis was restricted to a small, rather homogenous patient subset, without adjustment for multiple comparisons. Baseline characteristics were less balanced in the Japanese subgroup compared with the overall study population. As most Japanese patients treated with SC were aged > 65 years (87%) and received low-intensity chemotherapy (79%), a potential bias toward poorer outcomes with SC treatment in this Japanese subgroup cannot be ruled out. Thus, results from this analysis should be interpreted with caution.

Single-agent gilteritinib therapy improved response rates in Japanese patients with R/R *FLT3*^mut+^ AML and resulted in numerically longer survival compared with SC. The observed trends in treatment efficacy and safety are consistent with the overall ADMIRAL study population and confirm that gilteritinib is an important treatment option for the Japanese R/R *FLT3*^mut+^ AML population. Investigations of gilteritinib as part of induction/consolidation (NCT02236013 and NCT04027309) and as post-consolidation/post-HSCT maintenance therapy (NCT02927262 and NCT02997202) are ongoing.

## Data Availability

Researchers may request access to anonymized participant level data, trial level data and protocols from Astellas sponsored clinical trials at www.clinicalstudydatarequest.com. For the Astellas criteria on data sharing see: https://clinicalstudydatarequest.com/Study-Sponsors/Study-Sponsors-Astellas.aspx.
